# Loss of Dnmt3a induces CLL and PTCL with distinct methylomes and transcriptomes in mice

**DOI:** 10.1038/srep34222

**Published:** 2016-09-28

**Authors:** Staci L. Haney, Garland M. Upchurch, Jana Opavska, David Klinkebiel, Adams Kusi Appiah, Lynette M. Smith, Tayla B. Heavican, Javeed Iqbal, Shantaram Joshi, Rene Opavsky

**Affiliations:** 1 Department of Genetics, Cell Biology, and Anatomy, University of Nebraska Medical Center, Omaha, Nebraska, 68198, USA; 2 Eppley Institute for Research in Cancer and Allied Diseases, Fred and Pamela Buffett Cancer Center, University of Nebraska Medical Center, Omaha, Nebraska, 68198, USA; 3 Department of Biochemistry and Molecular Biology, University of Nebraska Medical Center, Omaha, Nebraska, 68198, USA; 4 College of Public Health, UNMC, Omaha, Nebraska, 68198, USA; 5 Department of Pathology and Microbiology, University of Nebraska Medical Center, Omaha, Nebraska, 68198, USA; 6 Center for Leukemia and Lymphoma Research, University of Nebraska Medical Center, Omaha, Nebraska, 68198, USA

## Abstract

Cytosine methylation of DNA is an epigenetic modification involved in the repression of genes that affect biological processes including hematopoiesis. It is catalyzed by DNA methyltransferases, one of which -DNMT3A- is frequently mutated in human hematologic malignancies. We have previously reported that Dnmt3a inactivation in hematopoietic stem cells results in chronic lymphocytic leukemia (CLL) and CD8-positive peripheral T cell lymphomas (PTCL) in *EμSRα-tTA;Teto-Cre;Dnmt3a*^*fl/fl*^*; Rosa26LOXP*^*EGFP/EGFP*^ (*Dnmt3a*^Δ/Δ^) mice. The extent to which molecular changes overlap between these diseases is not clear. Using high resolution global methylation and expression analysis we show that whereas patterns of methylation and transcription in normal B-1a cells and CD8-positive T cells are similar, methylomes and transcriptomes in malignant B-1a and CD8+ T cells are remarkably distinct, suggesting a cell-type specific function for Dnmt3a in cellular transformation. Promoter hypomethylation in tumors was 10 times more frequent than hypermethylation, three times more frequent in CLL than PTCL and correlated better with gene expression than hypermethylation. Cross-species molecular comparison of mouse and human CLL and PTCL reveals significant overlaps and identifies putative oncogenic drivers of disease. Thus, *Dnmt3a*^Δ/Δ^ mice can serve as a new mouse model to study CLL and PTCL in relevant physiological settings.

Cytosine methylation of DNA is an epigenetic modification affecting gene transcription and the integrity of the mammalian genome. Basic methylation patterns are established and maintained by catalytic activity of three DNA methyltransferases: DNMT1, DNMT3A, and DNMT3B. Promoter methylation is associated with transcriptional repression and plays a role in a variety of normal physiologic processes, including X-chromosome inactivation, genomic imprinting, differentiation and hematopoiesis^1^,^2^.

Non-Hodgkin’s lymphoma (NHL) is a heterogeneous group of lymphoid malignancies that arise from transformation of B, T, and NK cells. The majority of NHLs are B-cell lymphomas, the most common of which is chronic lymphocytic leukemia/small lymphocytic lymphoma (CLL/SLL): an indolent low-grade lymphoproliferation of mature B-cells^3^. However, T cell lymphomas develop in ~10% of NHL patients^4^. Approximately 30% of these T cell malignancies will be diagnosed as peripheral T cell lymphoma-not otherwise specified (PTCL-NOS): a group of high-grade mature T cell neoplasms not classified by other WHO criteria^5^. Both CLL and PTCL are life-threatening conditions that present in late adulthood and despite recent advances in chemotherapy, these diseases remain refractory to cure. A better understanding of deregulated molecular landscapes and a contribution of individual changes to the development of these two NHLs is needed to generate new therapeutic approaches.

Two types of molecular changes likely involved in the pathogenesis of CLL and PTCL are genetic alterations and epimutations such as de-regulated cytosine methylation. It was reported that an average of 45 somatic mutations are present in human CLL samples, with most genes mutated in less than 5% of cases^6^. About one third of cases did not have recurrent mutations, suggesting a high degree of heterogeneity and no clear genetic drivers of CLL. Interestingly, global gene expression profiling identified two DNA methyltransferases, Dnmt3b and Dnmt3a, as the top 1% of underexpressed genes in human CLL^7^, suggesting that the DNA methylation landscape may be deregulated. Indeed, DNA methylation profiling revealed a substantial genome wide promoter and gene-body hypomethylation in tumors relative to normal B cells^8^. Consistently with possible roles of Dnmt3a and Dnmt3b in CLL development we have previously reported that conditional inactivation of Dnmt3a in hematopoietic stem cells and progenitors in *EμSRα-tTA;Teto-Cre;Dnmt3a*^*fl/fl*^*;Rosa26 LOXP*^*EGFP/EGFP*^ (*Dnmt3a*^Δ/Δ^) mice resulted in the development of chronic lymphocytic leukemia (CLL) around 1 year of age and is accelerated when Dnmt3b is deleted as well^9^. Such data strongly suggest that Dnmt3a represses genes in normal B cells likely through promoter methylation whose up-regulation upon hypomethylation may contribute to the development of CLL.

The mutational landscape of T cell lymphoma (TCL) appears to be less diverse than in CLL. Interestingly, one of the most frequently mutated gene is DNMT3A, suggesting a possible involvement in disease development^10^. Although no comparable large scale profiling of the methylation landscape has been performed on TCL to date, our functional studies utilizing *EμSRα-tTA;Teto-Cre;Dnmt3a*^*fl/fl*^*;Rosa26 LOXP*^*EGFP/EGFP*^ mice demonstrated that Dnmt3a likely play a role in pathogenesis of PTCL. Although these mice primarily develop CLL, 30% of mice develop CD8-positive PTCL either in combination with CLL or by itself 9,11. However, the nature of deregulated events in both mouse diseases and how they relate to human diseases remains poorly understood.

To better understand the molecular changes occurring in Dnmt3a-deficient mice we performed global methylation profiling using whole genome bisulfite sequencing (WGBS) and gene expression profiling using RNA-seq on CLL and PTCL tumors isolated from *Dnmt3a*^Δ/Δ^ mice, as well as control B-1a and CD8+ T cells. This analysis revealed that while normal B-1a and CD8+ T cells had remarkably similar methylomes and transcriptomes, Dnmt3a loss induced unique changes in DNA methylation and gene transcription in CLL and PTCL. Importantly, analysis of available expression data from human CLL and PTCL samples, revealed a significant overlap between human and mouse transcriptomes and methylomes, suggesting *Dnmt3a*^Δ/Δ^ mice may serve as a useful tool to identify oncogenic drivers contributing to CLL and PTCL pathogenesis in humans.

## Results

### Cell-Autonomous Tumor Suppressor Function of Dnmt3a in the prevention of CLL

**Figure 1:**
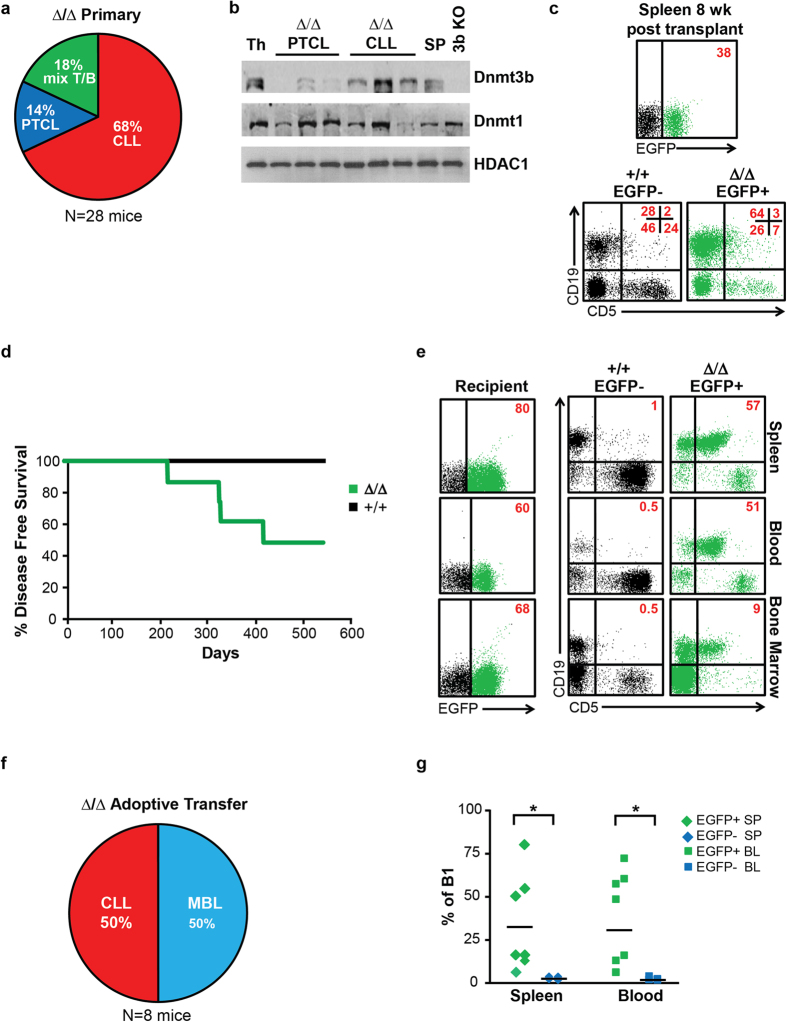
Dnmt3a’s tumor suppressor function is cell autonomous. **(a)** Percentage of *Dnmt3a*^Δ/Δ^ mice diagnosed with CLL (red), PTCL (blue), or mixed CLL/PTCL (green) at time of harvest, as determined FACS. N = 28. **(b)** Immunoblot analysis of Dnmt3b and Dnmt1 proteins in *Dnmt3a*^+/+^ normal thymus (Th) and spleen (SP), *Dnmt3a*^Δ/Δ^ PTCL, and *Dnmt3a*^Δ/Δ^ CLL samples. *Dnmt3b*^*−/−*^ (3b KO) cells were used as a negative control. HDAC1 is shown as a loading control. **(c)** Representative FACS diagram showing CD19 and CD5 expression in EGFP- (black) and EGFP+ (green) cellular populations isolated from the spleen of a lethally irradiated FVB recipient mouse injected with bone marrow *Dnmt3a*^Δ/Δ^ bone marrow. The mouse was harvested 8 weeks post injection. Percentage of cells in each quadrant are shown in the top right in red. **(d)** Kaplan-Meier survival curves for FVB mice lethally irradiated and injected with *Dnmt3a*^+/+^ *or Dnmt3a*^Δ/Δ^ (N = 8) bone marrow cells. **(e)** Representative FACS diagram showing CD19 and CD5 expression in EGFP- (black) and EGFP+ (green) cell isolated from the spleen, blood and bone marrow of a lethally irradiated FVB recipient mice injected with *Dnmt3a*^Δ/Δ^ bone marrow. The mouse was harvested 9 months post injection when terminally ill. Percentage of cells staining positive in each quadrant are shown in the top right in red. **(f)** Percentage of lethally irradiated wild-type mice injected with *Dnmt3a*^Δ/Δ^ bone marrow cell that were diagnosed with CLL (red) or MBL (blue) at time of harvest, as determined FACS. N = 8. **(g)** Percentage of EGFP+ (green) and EGFP- (blue) B-1a cells (B220 +CD19+ CD5+, determined by FACS) in the spleens and blood of lethally irradiated FVB recipient mice injected with *Dnmt3a*^Δ/Δ^ bone marrow. P < 0.05 is indicated by (*), two-tailed Student’s *t*-test.

To determine the role of Dnmt3a in hematopoiesis we used the *EμSRα-tTA;Teto-Cre;Dnmt3a*^*fl/fl*^*;Rosa26LOXP*^*EGFP/EGFP*^ (*Dnmt3a*^Δ/Δ^) mouse model to conditionally delete Dnmt3a in hematopoietic stem and progenitor cells (HSPCs) cells. In this system, deletion of Dnmt3a occurs in EGFP-positive cells (30–50% of all HSPCs) whereas EGFP-negative cells (50–70% of all HSPCs) retain both conditional Dnmt3a alleles (*Dnmt3a*^*fl/fl*^) and therefore behave like *wild-type* cells^9^. Using this model, we previously showed that a long-term Dnmt3a-defficiency resulted in the development of a chronic lymphocytic leukemia (CLL) in 68% of mice, CD8-positive mature T cell lymphomas (PTCL) in 14% mice and mixed CLL/PTCL in 18% cases within one year of age^9^,^11^. (Fig. 1a and data not shown).

To examine if the levels of other DNA methyltransferases were changed upon deletion of Dnmt3a, we analyzed protein levels of Dnmt1 and Dnmt3b in Dnmt3a-deficient PTCL and CLL samples. While we did not observe any consistent changes in the levels of Dnmt1 in either tumor type, we did see down regulation of Dnmt3b in PTCL, but not CLL samples, relative to their respective controls (Figs 1b and S1). Such molecular change may contribute to the pathogenesis of PTCL in particular because Dnmt3b loss accelerates MYC-induced lymphomagenesis as well as promotes the development of T cell lymphomas in *Dnmt3a*^Δ/Δ^ mice^9^,^12^.

To determine whether loss of Dnmt3a promotes tumorigenesis in a cell-autonomous way, we used adoptive transfer to introduce bone marrow cells isolated from 6 weeks old *Dnmt3a*^+/+^ control and *Dnmt3a*^Δ/Δ^ mice into lethally irradiated FVB recipient mice. Analysis of hematopoiesis in recipient mice revealed that both EGFP-negative and EGFP-positive cells contributed to reconstitution of hematopoietic system with no signs of disease 2 months after adoptive transfer (Fig. 1c and S2 and data not shown). Injected mice were observed for signs of tumorigenesis and harvested when terminally sick. Control mice remained disease free during the 500 days observational period (Fig. 1d). In contrast, 50% of *Dnmt3a*^Δ/Δ^ mice became terminally ill with CLL with a median survival of 311 days (Fig. 1d–g). The remaining 50% of *Dnmt3a*^Δ/Δ^ mice developed monoclonal B cell lymphopoiesis (MBL), a less advanced form of CLL, in which the percentage of B-1a cells in the blood ranges from 2–20% (Fig. 1d–g). EGFP-positive CLL or MBL splenic cells induced disease in sublethally irradiated secondary FVB recipients, suggesting that tumors contain leukemia-initiating cells (data not shown). Altogether, these results show that the Dnmt3a’s tumor suppressor function in prevention of CLL resides within the hematopoietic compartment and is likely cell-autonomous. Due to the lower frequency of PTCL development in Dnmt3a-deficient mice, larger cohorts of mice will need to be analyzed to determine whether Dnmt3a tumor suppressor function in prevention of PTCL is also autonomous to hematopoietic system.

### DNA methylome and transcriptome is highly similar between normal mouse B-1a and CD8 cells

**Figure 2:**
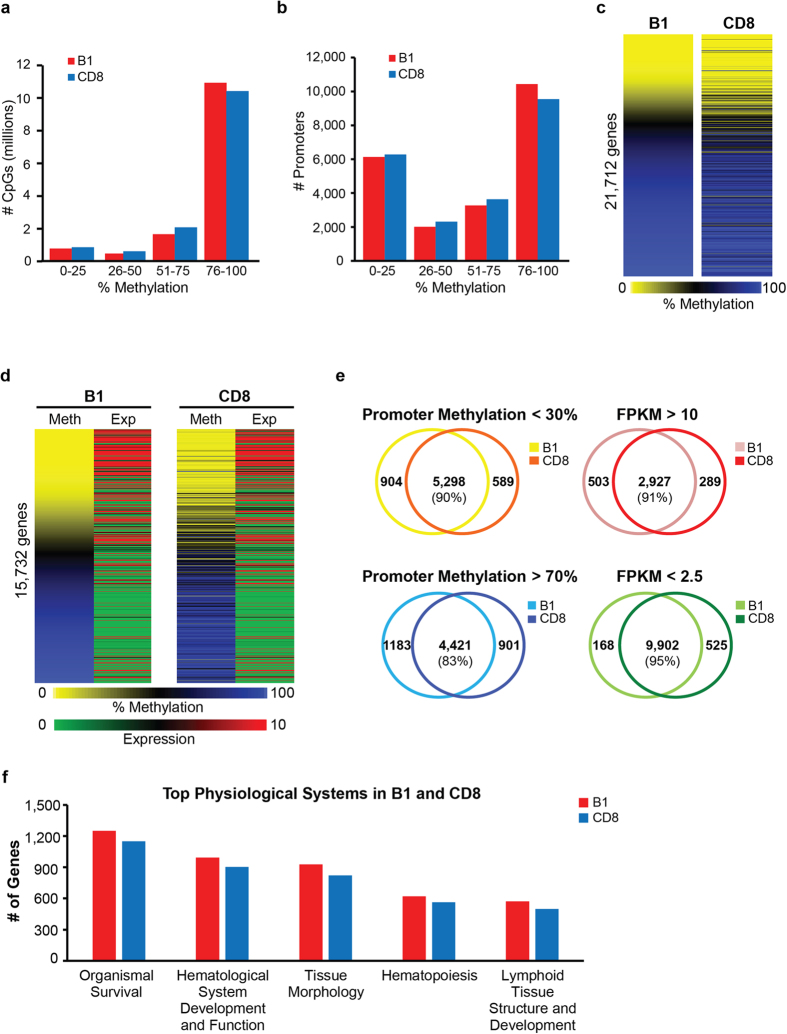
Transcriptome and methylome of normal B-1a and CD8 cells. **(a)** Breakdown of CpG methylation as determined by WGBS in B-1a (B1) and CD8 samples. Individual CpGs were placed into 4 categories based on percent methylation (0–25%, 26–50%, 51–75%, and 76–100%). **(b)** Breakdown of promoter methylation for 21,712 genes in B1 and CD8 samples. Methylation percentage for individual CpGs were annotated to the core promoter regions (−300 bp to +150 bp relative to the TSS). Methylation percentages for all CpGs across the 450 bp region were averaged to give a mean methylation value for each gene promoter. Promoters were placed in 4 categories based on percent methylation (0–25%, 26–50%, 51–75%, and 76–100%). **(c)** Methylation status of 21,712 promoters in B1 and CD8 samples as determined by WGBS. Mean promoter methylation was determined as described in part b of the figure legend. Hypomethylation is shown in yellow and hypermethylation in blue. **(d)** Heat map presentation of promoter methylation (analyzed as in Fig. 2b) and corresponding gene expression (presented as average FPKM values as determined by RNA-seq) in mouse splenic B1 and CD8 cells for 15,732 genes. Genes with high FPKM values are shown in red and genes with low FPKM values are shown in green. Heat maps are organized in the same gene order to match data for methylation and gene expression. **(e)** (left) Venn diagram showing number of unique and overlapping hypomethylated (methylation <30%) and hypermethylated (methylation >70%) promoters in B1 and CD8 samples. (right) Venn diagram showing number of unique and overlapping highly expressed (FPKM > 10) and lowly expressed (FPKM <2.5) genes in B1 and CD8 samples. **(f)** Ingenuity Pathway analysis (IPA) of highly expressed genes (FPKM ≥10) in B1 (red) and CD8 (blue) samples. The top subcategories obtained in Physiological System, Development and Functions are displayed (P < 0.05, for all subcategories).

To determine the nature of deregulated molecular events during CLL and PTCL development in *Dnmt3a*^Δ/Δ^ mice we performed global methylation analysis by Whole Genome Bisulfite Sequencing (WGBS) and gene expression profiling by RNA-seq on B220+ CD19+ CD5+ (B-1a cells) and CD8-positive T cells isolated from *Dnmt3a*^+/+^ spleens, as these cellular populations are immunophenotypically the closest normal counterparts of CLL and PTCL cells, respectively. Analysis of methylation in 15,533,510 matched CpG dinucleotides distributed across the genome revealed a high degree of methylation in both cell types, with higher levels of methylation in B-1a cells. For example, more CpG dinucleotides were methylated at levels ≥76% in B- than in T-cells (79% and 75% in B-1a and CD8+ T cells, respectively) (Fig. 2a). In contrast, fewer CpG dinucleotides were methylated at low levels (≤25%) in B- than T- cells (5.5% and 6% in B-1a and CD8+ T cells, respectively). Increased methylation in B-1a cells relative to CD8+ T cells was also detected in core promoters. More promoters were methylated at levels ≥76% in B- than T-cells (48% and 44% in B-1a and CD8+ T cells, respectively) (Fig. 2b). A smaller number of promoters was methylated at low levels (≤25%) in B- than in T- cells (28% and 29% in B-1a and CD8+ T cells, respectively) (Fig. 2b). Methylation of 21,712 core promoters (−300+ 150 bp relative to TSS) was remarkably similar on a locus specific level between these two normal cell types, although some promoters were differentially methylated in B-1a B cells and CD8+ T cells (Figs 2c and S3, Supplemental Table S1). A combined gene expression and methylation analysis revealed that the majority of genes with low levels of promoter methylation were expressed, whereas genes with high levels of promoter methylation were largely repressed (Figs 2d and S4, Supplemental Table S2). Promoter methylation was well conserved between B-1a and CD8+ cells, with 90% of hypomethylated and 83% of hypermethylated promoters common between B-1a and CLL (Fig. 2e). Similarly, more than 90% of highly and lowly expressed genes were shared between B-1a and CD8+ cells (Fig. 2e). Ingenuity pathway analysis (IPA) of highly expressed genes in B-1a and CD8 cells revealed the same top subcategories of genes significantly associated with *organismal survival*, *hematologic system*, *tissue morphology*, *hematopoiesis*, *lymphoid tissue structure* (Figs 2f and S5), highlighting a large overlap in genes commonly expressed in B1a and CD8 cells, as well as their link to hematopoietic system. However, IPA analysis of genes only overexpressed in individual cell types (503 and 289 genes in B-1a and CD8, respectively) revealed specific differences. For example, genes associated with categories such as *proliferation of B lymphocytes, quantity of B lymphocytes* or *quantity of IgG* were only detected using data obtained from B-1a cells (Fig. S6). Similarly, categories such as *quantity of CD8+ T lymphocyte*, *activation of T lymphocytes* or *morphology of thymus gland* were only detected using data obtained from CD8 cells (Fig. S6). Altogether, these data show large scale hypermethylation in both normal cell types, with B-1a cell showing higher levels of methylation. In addition, methylation and gene expression in normal splenic B-1a cells and CD8 cells is largely shared with the exception of a subset of genes specifically involved in processes associated with unique functions of these cell types.

### ***Dnmt3a*** ^Δ/Δ^ CLL and ***Dnmt3a*** ^Δ/Δ^ PTCL have distinct methylomes

**Figure 3:**
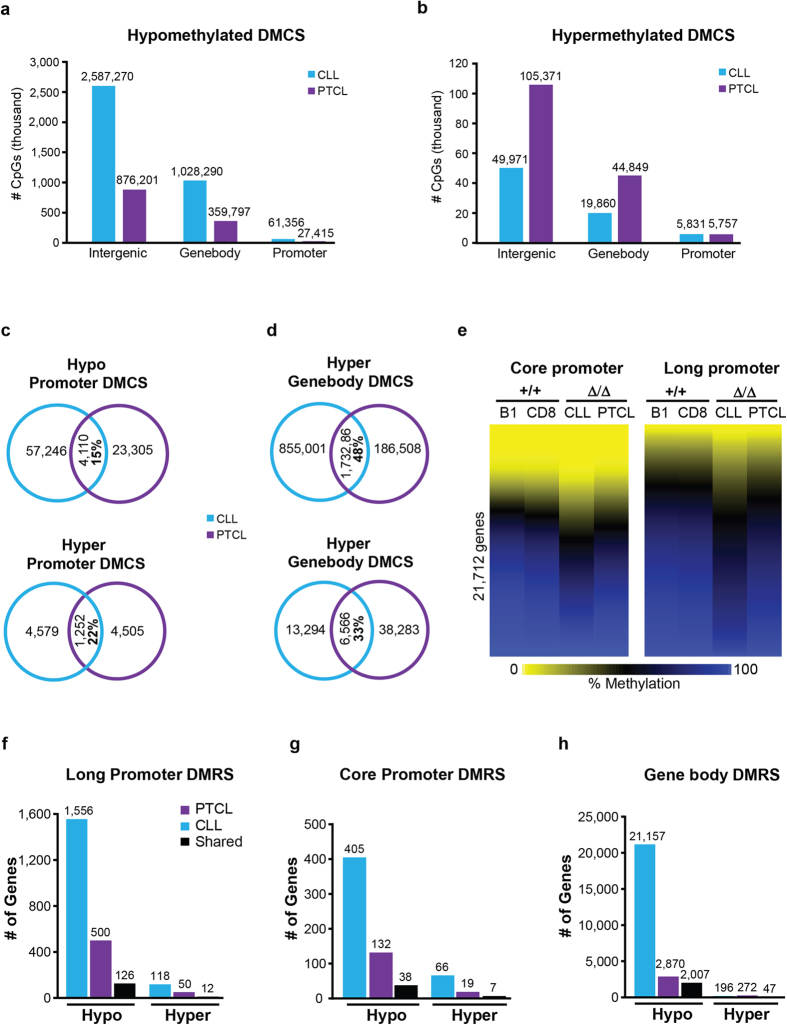
DNA methylome of *Dnmt3a*^Δ/Δ^ CLL and PTCL. **(a)** Breakdown of hypomethylated and **(b)** hypermethylated CpGs (differentially methylated CpGs; DMCS) in *Dnmt3a*^Δ/Δ^ CLL (relative to B-1a) and PTCL (relative to CD8) categorized by genomic location. Differentially methylated CpGs (defined as a methylation difference of ≥30%) were annotated to gene promoters (−1,500 to +500 bp relative to TSS), gene-bodies, or intergenic regions. **(c)** Venn diagram showing unique and overlapping hypomethylated (methylation <30%) and hypermethylated (methylation >70%) CpGs annotated to long promoters (−1,500 to +500 bp relative to TSS) and **(d)** gene-bodies in CLL (blue) and PTCL (purple). **(e)** Heat map displaying the methylation status of 21,712 promoters as determined by WGBS in B1, CD8, *Dnmt3a*^Δ/Δ^ CLL, and *Dnmt3a*^Δ/Δ^ PTCL. Methylation percentage for individual CpGs were annotated to the core promoter regions (−300 bp to +150 bp relative to the TSS, *left*) and long promoter regions (−300 bp to +150 bp relative to the TSS, *right*). Methylation percentages for all CpGs across the region were averaged to give a mean methylation value for each gene promoter. Hypomethylation is shown in yellow and hypermethylation in blue. **(f)** The number of genes with differentially methylated regions (DMRS) in their long promoters, **(g)** core promoters and **(h)** gene-bodies in *Dnmt3a*^Δ/Δ^ PTCL (purple) relative to CD8 control, and *Dnmt3a*^Δ/Δ^ CLL (blue) relative to B-1a control. The DMRS shared between tumor types are shown in black.

To determine the effects of loss of Dnmt3a on cancer methylomes we next performed WGBS on DNA isolated from *Dnmt3a*^Δ/Δ^ CLL and PTCL cells. Out of ~14 million CpG dinucleotides analyzed, we observed decreased methylation in 3,676,916 (26.5%) CpGs and increased methylation in 75,662 (0.5%) of CpGs in CLL relative to B-1a (Fig. S7). Although methylation changes in PTCL were less pronounced than in CLL, hypomethylation was still the major change, as decreased methylation was observed in 1,263,413 (9%) CpGs and increased methylation in 155,977 (1%) CpGs (Fig. S7). Unsupervised hierarchical cluster analysis using methylation readouts for all individual CpGs revealed tight clustering of normal cells, whereas tumors were both significantly distinct from both their normal counterparts and each other (Fig. S8). Although the vast majority of changes in methylcytosine levels occurred in gene bodies and intergenic regions in both disease settings, we also observed significant changes in promoter methylation (Fig. 3a). Interestingly, the number of hypomethylated cytosines in promoters was almost 2.2 fold higher in CLL (61,356) than in PTCL (27,415) (Fig. 3a). In contrast, the number of hypermethylated cytosines in promoters were almost equal between CLL (5,831) and PTCL (5,757) (Fig. 3b). Notably, a relatively small number of cytosines associated with long promoters (−1,500 to +500 bp relative to TSS) were commonly hypo- and hypermethylated in CLL and PTCL (15% and 22%, respectively; Fig. 3c and Supplemental Table S3). In contrast, differentially methylated cytosines in gene-bodies were more commonly shared between the two disease settings (48% hypomethylated and 33% hypermethylated CpGs Fig. 3d). Consistent with the greater number of hypomethylated CpGs observed in CLL, analysis of differentially methylated regions revealed that the number of hypomethylated promoters was ~ 3-fold higher in CLL relative to PTCL in both long and core (−300 to +150 relative to TSS) promoters (Fig. 3e–g and Supplemental Table S4). Interestingly, only 126 out of 500 long promoters hypomethylated in PTCL were also hypomethylated in CLL and this trend could also be seen in core promoters (Fig. 3g and Supplemental Table S5). The difference in gene body hypomethylation was even more pronounced with more than 7-fold more genes affected in CLL than in PTCL (Fig. 3h and Supplemental Table S5). These data suggest that Dnmt3a may play broader functions in promoter and gene body methylation in B cells than in T cells. In addition to hypomethylation we also observed promoter and gene body hypermethylation in CLL and PTCL (Fig. 3f–h). Like with hypomethylation, most hypermethylation events were tumor type specific.

Analysis of the spatial distribution of DNA methylation changes revealed that areas around the TSS were –not surprisingly – the least methylated areas in both normal and tumor cells (Fig. S9). Consistently with analysis of individual elements, the degree of hypomethylation was higher in CLL than in PTCL, with no substantial differences observed in the spatial distributions of methyl cytosines across gene elements (Fig. S9).

**Figure 4:**
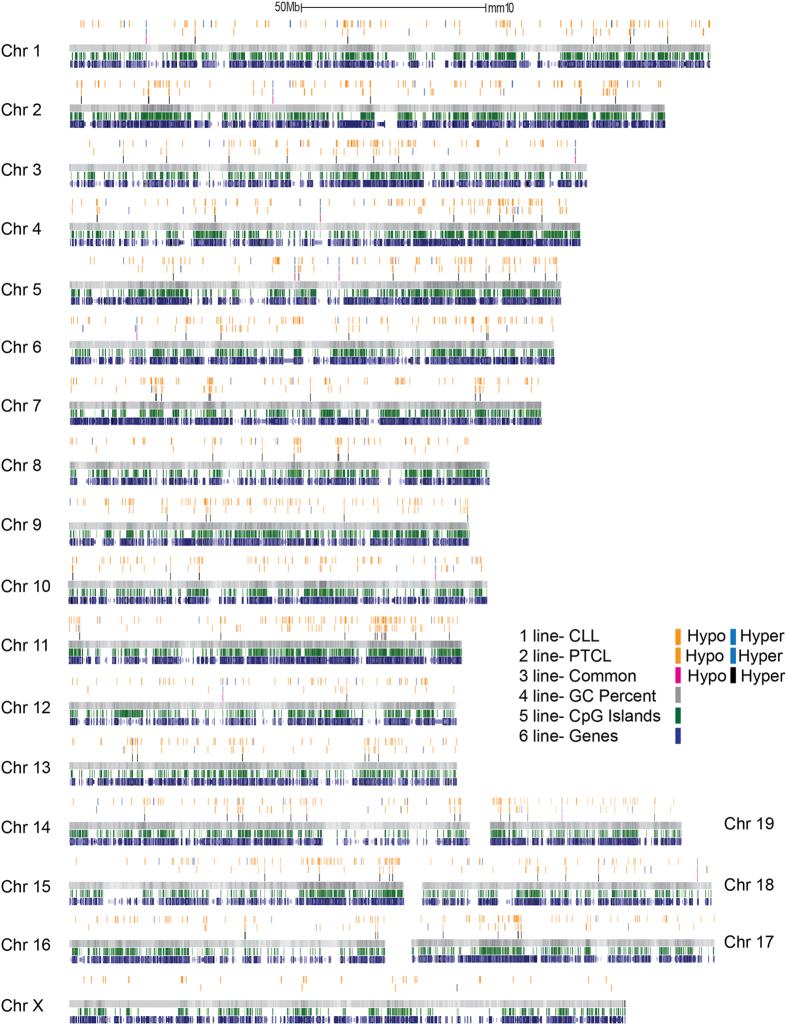
Loss of Dnmt3a results in unique methylation patterns in *Dnmt3a*^Δ/Δ^ CLL and PTCL. Chromosome plot of differentially methylated regions annotated to the promoter region (−1,500 to +500 bp relative to TSS) in *Dnmt3a*^Δ/Δ^ CLL (1^st^ row, orange bar) relative to B-1a and *Dnmt3a*^Δ/Δ^ PTCL (2^nd^ row, purple bar) relative to CD8 control. Hypomethylated DMRS are indicated by orange lines and hypermethylated DMRS by blue. DMRS that are shared between the two tumor types are also shown (3^rd^ row, red bar). The CG percentage (4^th^ row, gray bar), location of CpG islands (5^th^ row, green bar) and location of genes (6^th^ row, blue bar) are also shown. 50Mb scale is shown to reference chromosome length.

Locus-specific analysis revealed that hypo- and hypermethylated promoters were relatively equally distributed across the genome, with exception of the X chromosome in which very few differentially methylated promoters were detected in CLL or PTCL (Fig. 4). The highest number of hypomethylated promoters in CLL were present on chromosomes 11 and 9, whereas in PTCL chromosomes 11 and 5 were most affected (Fig. 4). Like in human CLL^8^, large scale gene body hypomethylation (21,157 genes) was observed in the CLL genome, along with hypermethylation of 196 genes. The trend was similar, but less pronounced in the PTCL methylome, with little overlap between tumor types (Fig. 3h).

**Figure 5:**
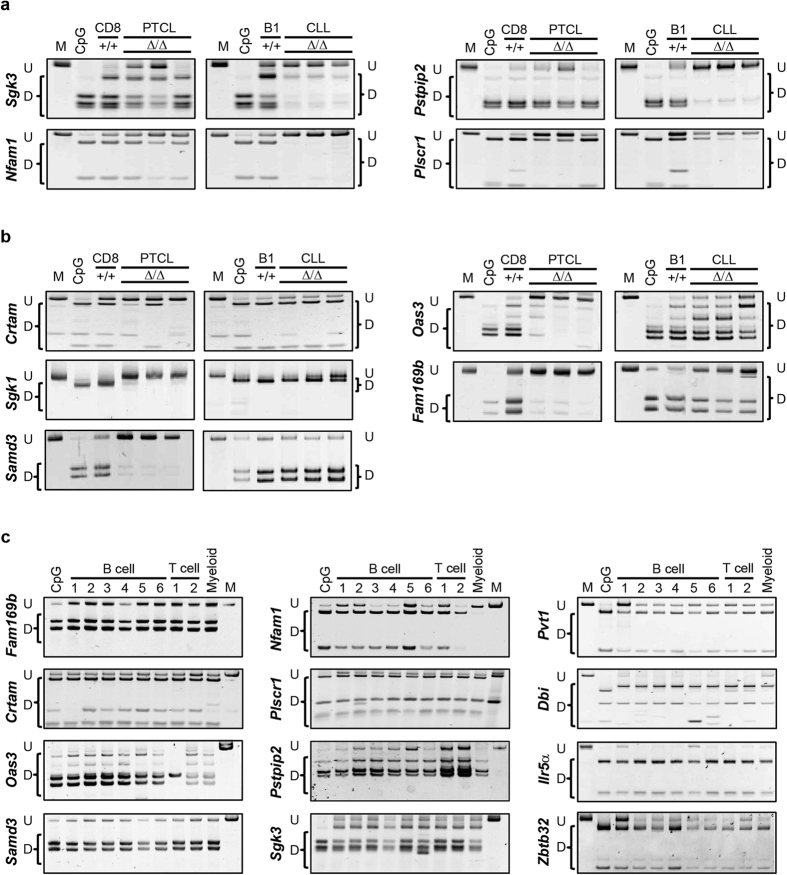
Promoter hypomethylation in *Dnmt3a*^Δ/Δ^ CLL and PTCL is common in additional tumors. COBRA analysis of promoter methylation for **(a)** *Sgk3, Nfam1, Pstpip2, Plscr1* and **(b)** *Crtam, Sgk1, Samd3, Oas3 and Fam169b* in *wild-type* CD8+ T cells, *wild-type* B-1a cells, *Dnmt3a*^Δ/Δ^ PTCL and *Dnmt3a*^Δ/Δ^ CLL samples. PCR fragments amplified from bisulfite-treated genomic DNA were digested with the restriction enzymes *BstU*I, *Taq*1 or *Tai*I. Undigested (U) and digested (D) fragments correspond to unmethylated and methylated DNA, respectively. Control PCR fragments generated from fully methylated mouse genomic DNA that is undigested (M) or digested (CpG) are shown. **(c)** COBRA analysis of promoter methylation for *Fam169b, Crtam, Oas3, Samd3, Nfam1, Plscr1, Pstpip2, Shk3, Pvt1, Dbi, Il5ra*, and *Zbtb32.* The following samples were isolated from *wild-type* mice, B cell subsets: (1) splenic B-1a, (2) bone marrow immature B cells, (3) bone marrow mature B cells, (4) splenic CD19+ B cells, (5) splenic marginal zone B cells, (6) splenic follicular B cells, T cell subsets: (1) splenic CD4+ T cells, (2) splenic CD8 T cells and lastly CD11b+ (Myeloid) splenic cells.

To further validate data obtained by global methods we performed locus-specific methylation analysis using Combined Bisulfite Restriction Analysis (COBRA) on promoters specifically hypomethylated in either CLL or PTCL. Similar to WGBS, promoters of *Sgk3, Nfam1, Pstpip2, and Plscr1* were found to be hypermethylated in normal B-1a cells, normal CD8 T cells, and *Dnmt3a*^Δ/Δ^PTCL, but were hypomethylated in three independent *Dnmt3a*^Δ/Δ^ CLL samples (Fig. 5a). We also confirmed that the promoters of *Crtam, Sgk1, Samd3, Oas3*, and *Fam169b* were hypermethylated in normal B-1a, normal CD8 T cells, and *Dnmt3a*^Δ/Δ^CLL, but were hypomethylated in *Dnmt3a*^Δ/Δ^PTCL (Fig. 5b). Overall, our data demonstrate that changes in promoter methylation identified using WGBS likely represent common events occurring in mouse CLL and PTCL and demonstrate the cell-type specific patterns induced by loss of Dnmt3a.

To determine if promoter hypomethylation is cancer-specific or is present in other normal cell types, we evaluated the methylation status of promoters in a range of B cell subsets (immature B cells, mature B cells, B-1a, CD19+, follicular B cells, and marginal zone B cells), T cells (CD4 and CD8), and myeloid cells. Interestingly, for the majority of genes analyzed, the promoters were heavily methylated in all normal cell types (Fig. 5c) suggesting that promoter hypomethylation does not occur during differentiation but it rather is tumor-type specific event. Altogether, our data suggest that Dnmt3a may have cell-type specific functions in maintaining promoter and gene-body methylation that seems to be more pronounced in B-1a cells than in CD8+ cells, but which may nonetheless be critical to prevent transformation of both normal cell types.

### ***Dnmt3a*** ^Δ/Δ^ CLL and ***Dnmt3a*** ^Δ/Δ^ PTCL have distinct transcriptomes

**Figure 6:**
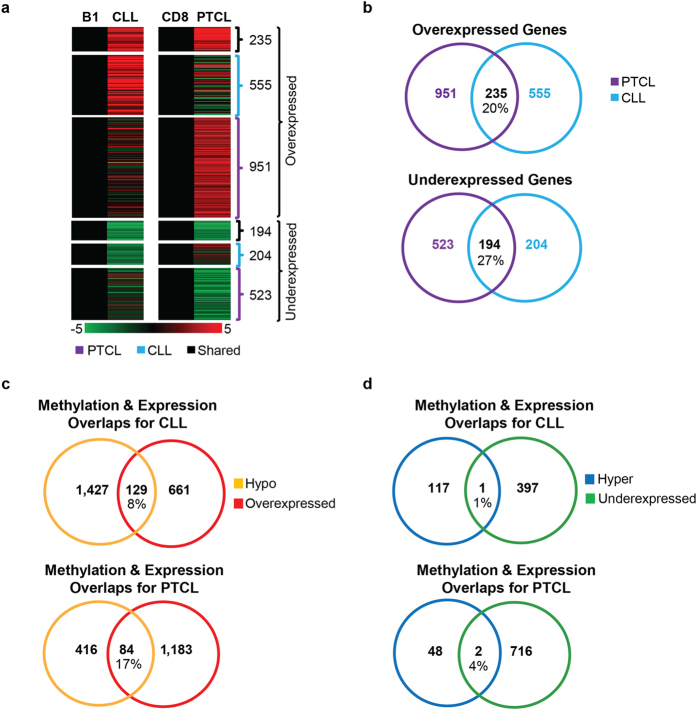
Gene expression is deregulated in a cell-specific manner in *Dnmt3a*^Δ/Δ^ CLL and PTCL. **(a)** Heat maps derived from global expression profiling by RNA-seq. 555 Genes were overexpressed exclusively in *Dnmt3a*^Δ/Δ^ CLL (N = 8) relative to B-1a (B1) controls (N = 2), 951 genes were overexpressed exclusively in *Dnmt3a*^Δ/Δ^ PTCL (N = 3) relative to CD8 controls (N = 2), and 235 genes were overexpressed in both tumor types. 204 Genes were underexpressed exclusively in *Dnmt3a*^Δ/Δ^ CLL relative to B1 controls, 523 genes were underexpressed exclusively in *Dnmt3a*^Δ/Δ^ PTCL relative to CD8 controls, and 194 genes were underexpressed in both tumor types. A color bar is shown to reference fold change with upregulation in red and downregulation in green. Only genes with a fold change ≥2 and a q-value <0.05 are shown. **(b)** Venn diagram showing the number of unique and overlapping differentially expressed genes in CLL (blue) and PTCL (purple). **(c,d)** A graphical presentation of the number of genes differentially expressed (red; overexpression, green; underexpression) and the number of genes differentially methylated at the promoter region (yellow; hypomethylation, blue; hypermethylation) in *Dnmt3a*^Δ/Δ^ CLL relative to B1 controls and in *Dnmt3a*^Δ/Δ^ PTCL relative to CD8 controls. The number of genes with corresponding methylation and expression changes (Hypomethylated and overexpressed; top and hypermethylated and underexpressed; bottom) are shown in the overlapping portion of the circles.

Next, we performed RNA-seq on *Dnmt3a*^Δ/Δ^PTCL and CLL to identify genes differentially expressed relative to CD8+ and B-1a cells, respectively. As with methylation, expression patterns in CLL and PTCL were dramatically different with only 235 upregulated (20%) and 194 downregulated (27%) genes common between the two tumor types (Fig. 6a,b and Supplemental Table S6). *Dnmt3a*^Δ/Δ^CLL had 555 upregulated and 204 downregulated genes that were not common to PTCL, while *Dnmt3a*^Δ/Δ^PTCL had 951 uniquely upregulated and 523 downregulated genes (Fig. 6a,b and Supplemental Table S6). Ingenuity pathway analysis (IPA) using all gene expression changes found in tumors did not provide a clear clue to the pathways promoting CLL or PTCL development resulting in many pathways being activated or inhibited in *Dnmt3a*^Δ/Δ^ diseases (Fig. S10). Comparison of promoter methylation and gene expression revealed that 129 genes (8%) whose promoters were hypomethylated in CLL were associated with overexpression (Figs 6c and S11 referred to herein as HOC genes–Hypomethylated and overexpressed in CLL). A similar comparison of promoter methylation and gene expression revealed that 84 genes (17%) whose promoters were hypomethylated in PTCL were associated with overexpression (Figs 6c and S12, referred to herein as HOT genes–Hypomethylated and overexpressed in TCL). In contrast, we detected only two genes for PTCL and one gene for CLL whose hypermethylation correlated with underexpression, suggesting that most of the cancer-specific hypermethylation has little effect on gene expression and tumor progression (Fig. 6d). Altogether, these data demonstrate that hypomethylation affects gene expression on a broader scale than hypermethylation in mouse *Dnmt3a*^Δ/Δ^ CLL and PTCL.

### Identification of genes commonly overexpressed in mouse and human CLL and PTCL as potential drivers of disease development

**Figure 7:**
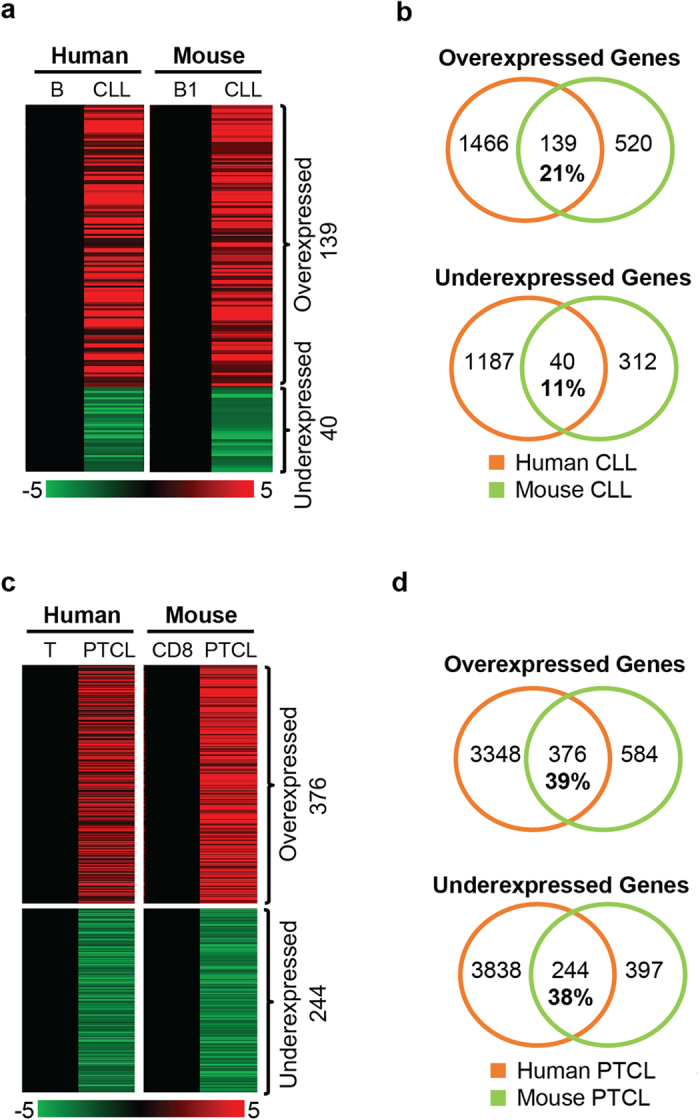
Gene expression is partially conserved between mouse and human CLL and PTCL. **(a)** Heat maps derived from global expression profiling by RNA-seq for human and mouse CLL. 139 genes were commonly overexpressed while 40 genes were commonly underexpressed in human CLL relative to CD19+ B cells and mouse *Dnmt3a*^Δ/Δ^ CLL relative to B1-a controls. Only genes with a fold-change >2 and q-value <0.05 were considered differentially expressed. **(b)** Venn diagrams showing overlaps in gene expression between human and mouse CLL datasets, as determined in panel a. **(c)** Heat maps derived from global expression for human PTCL (determined by microarray) and mouse PTCL (determined by RNA-seq). 376 genes were commonly overexpressed, while 244 genes were commonly underexpressed between human PTCL relative to normal tonsil T cells and mouse *Dnmt3a*^Δ/Δ^ PTCL relative to CD8 controls. For microarray data, genes with a fold change >1.5 and a P-value <0.05 were considered significant. For RNA-seq data, genes with a fold change >2 and a q-value <0.05 were considered significant. **(d)** Venn diagrams showing overlaps in gene expression between human and mouse PTCL datasets, as determined in panel c.

To determine the extent of similarity between mouse and human disease on the molecular level, we compared gene expression signatures obtained from mouse CLL and PTCL to the corresponding human disease. We utilized RNA-seq data obtained on a set of 10 human CLL samples^13^, in which 1,605 genes were up- and 1,227 genes were down-regulated relative to normal CD19+ B cells (not shown). When we compared expression of these genes to those commonly deregulated in mouse *Dnmt3a*^Δ/Δ^ CLL we found, we found a 21% overlap (139/659) between overexpressed genes and an 11% (40/352) overlap for underexpressed genes (Fig. 7a,b and Supplemental Table S7). The extent of overlap in up-and downregulated genes was significant for both comparisons (P < 0.00001 and P < 0.00003, respectively), suggesting that similar molecular events may drive CLL in both species.

Like for CLL, we were interested in determining the extent of overlap between genes deregulated in mouse and human PTCL. Therefore, we analyzed genes commonly up- and down-regulated in mouse *Dnmt3a*^Δ/Δ^ PTCL and human PTCL. As a source of human gene expression we utilized data obtained by microarray on a set of 15 PTCL samples and 5 normal T cell samples^10^,^14^. Our analysis revealed that 39% of genes (376/960) overexpressed in mouse PTCL were also overexpressed in human PTCL samples (Fig. 7c,d and Supplemental Table S7). Similarly, 38% of genes (244/641) under-expressed in mouse PTCL were also under-expressed in human PTCL samples. Like in CLL, the extent of overlap in up-and down-regulated genes was significant (P < 0.00001 for both comparisons), suggesting that similar molecular events may drive PTCL in both species.

**Figure 8:**
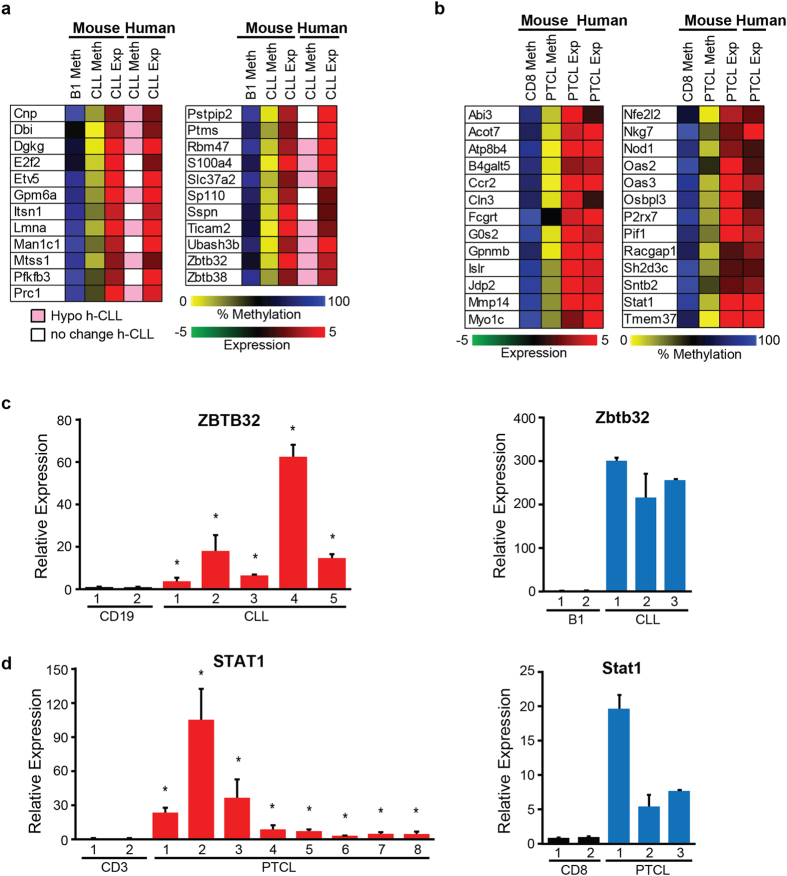
Identification of genes hypomethylated and overexpressed in mouse and human CLL and PTCL. **(a)** Heat map showing genes that were identified as hypomethylated and overexpressed in *Dnmt3a*^Δ/Δ^ CLL relative to B1-a controls (B1) that were also overexpressed in human CLL samples, as determined by RNA-seq. Genes that were identified to be hypomethylated at their promoters in human CLL are shown in pink, while those with no-change in promoter methylation are shown in white. Hypomethylation is shown in yellow and hypermethylation in blue, while overexpression is shown in red. **(b)** Heat map showing genes that were identified as hypomethylated and overexpressed in *Dnmt3a*^Δ/Δ^ PTCL relative to CD8 controls that were also overexpressed in human PTCL samples, as determined by microarray. Hypomethylation is shown in yellow and hypermethylation in blue, while overexpression is shown in red. **(c)** Real-time qRT-PCR analysis of ZBTB32 expression in human CD19 controls and CLL samples and analysis of STAT1 expression in human CD3 controls and PTCL samples. Averages of two independent experiments are presented. (*) denotes P < 0.05 (two-tailed student’s t-test) with error bars representing 1 Stdev. **(d)** Real-time qRT-PCR analysis of Zbtb32 expression in mouse B1 controls and *Dnmt3a*^Δ/Δ^ CLL samples and analysis of Stat1 expression in mouse CD8+ T cell controls and *Dnmt3a*^Δ/Δ^ PTCL samples. Averages of two independent experiments are presented. (*) denotes P < 0.05 (two-tailed student’s t-test) with error bars representing 1 Stdev.

To gain further insight into genes that may drive CLL and PTCL we considered the simplest explanation for the tumor suppressor function of Dnmt3a in the prevention of mouse CLL and PTCL - loss of methylase activity of Dnmt3a results in hypomethylation and overexpression of subset of genes whose inappropriate expression contributes to cellular transformation. We identified 129 and 84 genes hypomethylated and overexpressed in CLL (HOC genes) and PTCL (HOT genes), respectively (Fig. S12). In CLL, 23 HOC genes were overexpressed in human tumors, with 14 reported to be hypomethylated^8^ (Fig. 8a). Similarly, 26 HOT genes were overexpressed in human CD8+ PTCL but their methylation status have not been reported yet Fig. 8b. Using human CLL and PTCL samples, we confirmed the overexpression of two genes from the HOT and HOC signature. In particular, *Zbtb32* (Zinc Finger And BTB Domain Containing gene) was highly up-regulated in both human and mouse tumors relative to normal controls, suggesting that its increased expression is a common event in human and mouse CLL (Fig. 8c). Similarly, *Stat1* (Signal Transducer And Activator Of Transcription 1) was highly up-regulated in both human and mouse PTCL relative to normal CD3+ T cells, suggesting that its increased expression is a common event in human PTCL (Fig. 8d). Altogether, these data suggest that mouse and human CLL and PTCL could have common drivers of disease development and that the *EμSRα-tTA;Teto-Cre;Dnmt3a*^*fl/fl*^*;Rosa26LOXP*^*EGFP/EGFP*^model is a suitable model to study pathology of CLL and PTCL.

## Discussion

DNA methylation is an important mechanism of transcriptional regulation that plays a role in the proper differentiation of hematopoietic stem cells into differentiated lineages^2^,^15^. In cancer, the key methylation event contributing to tumorigenesis is considered to be promoter hypermethylation, which results in the inactivation of a wide range of tumor suppressor genes such as VHL, p16 and MLH1^16^. However, emerging evidence suggest that inactivation of methylase activity promotes the development of hematologic malignancies and is almost uniformly accompanied by large scale hypomethylation^9^,^11^,^12^,^17^,^18^,^19^,^20^. Such hypomethylation is not limited to non-coding areas of the genome but also affects a large number of promoters and gene bodies. As a result, the untimely expression of genes normally silenced may contribute to cellular transformation of normal cells. Thus, to begin to understand what molecules may be responsible for the transformation of normal cells, high resolution genome-wide approaches are needed to profile both normal and tumor cells to identify the nature of deregulated events. Here we used WGBS coupled with RNA-seq to determine the extent to which two malignancies that develop in the absence of Dnmt3a, CLL and PTCL, share deregulated molecular events. Analysis of promoter methylation revealed conservation between 90% of hypomethylated and 83% of hypermethylated promoters in B-1a B cells and CD8+ T cells. Similarly, gene expression was highly alike between these two cell types, with 91% and 95% of highly and lowly expressed genes overlapping, respectively. These results suggest a high level of similarities between normal methylomes and transcriptomes of B-1a and CD8+ T cells. These results are in stark contrast to malignant B-1a and CD8+ cells. Only 8% of hypomethylated promoters detected in CLL were also hypomethylated PTCL. Similarly, only 10% of hypermethylated promoters were shared between the two tumor types. Consistently, the transcriptomes of CLL and PTCL were substantially different, sharing only 20% of overexpressed and 27% of underexpressed genes. We further show that hypomethylation correlates with increased gene expression in 8% genes in CLL and 17% genes in PTCL. In contrast, hypermethylation correlated with gene underexpression in 1% and 4% in CLL and PTCL, respectively. Finally, we show that 21% of overexpressed and 11% of underexpressed genes are conserved between mouse and human CLL. The degree of conservation is higher between mouse and human PTCL with 39% of up-regulated and 38% of down-regulated shared between mouse and human PTCL. These data along with downregulation of DNMT3A in human CLL and mutations detected in human PTCLs suggest that *EμSRα-tTA;Teto-Cre;Dnmt3a*^*fl/fl*^*;Rosa26LOXP*^*EGFP/EGFP*^ can serve as a new mouse model to study CLL and PTCL in mice.

Several interesting observations can be made from data presented in this study. First, our data suggest that despite the similar methylomes and transcriptomes of normal B-1a and CD8 cells, Dnmt3a-deficient T- and B-cell malignancies are characterized by highly cell-type specific changes in both methylation and transcription. We found that the extent of promoter hypomethylation is ~3-fold higher in malignant B-1a cells than in malignant CD8 cells relative to their normal counterparts. This promoter hypomethylation either results from a failure to maintain established methylation patterns during tumor progression due to a lack of Dnmt3a or from an increased proliferation of cancer cells resulting from transformation but not necessarily linked to Dnmt3a maintenance activity. The reasons for cancer-specific patterns evolving in the absence of Dnmt3a remains unclear. It is possible that methylation of the same loci is controlled by different Dnmts or their complexes in B-1a and CD8 cells.

Relative abundance of accessory factors such as Dnmt3L or acquisition of genetic alterations affecting evolution of the epigenome may also differentially affect locus-specific methylation. It is also possible that some hypomethylated loci in tumors are already present in normal *Dnmt3a*^Δ/Δ^ B-1a and CD8 cells as a result of the lack of *de novo* methylation performed by Dnmt3a during normal differentiation. Finally, the ability of B-1a to self-renew may also contribute to accelerated hypomethylation and might be a reason why B-1a cells are preferentially transformed over other hematopoietic cell types in the absence of Dnmt3a. Cell-type specific methylation patterns between acute myeloid leukemia (AML), myelodysplastic syndrome (MDS), and CD4+ CD8+ T cell acute lymphoblastic leukemia (T-ALL) that develop in *Mx-1-Cre;Dnmt3a*^*fl/fl*^ mice have been previously reported. Mayle and colleagues observed that while methylation changes that arose in AML and MDS overlapped significantly, T-ALL was characterized by unique methylation patterns. Interestingly, the major methylation change observed in *Mx-1-Cre;Dnmt3a*^*fl/fl*^ mice with T-ALL was hypermethylation, with approximately 75% of DMRS showing a gain in methylation in tumors relative to controls. This is in strong contrast to our data in which hypomethylation was the dominant event in both PTCL and CLL induced by loss of Dnmt3a^18^.

A second important question raised by this study is the extent to which *Dnmt3a*^Δ/Δ^ mouse CLL and PTCL models are similar to human disease. The development of CLL in the absence of Dnmt3a is consistent with findings that Dnmt3a belongs to the top 1% of underexpressed genes in human CLL^7^,^9^. The development of PTCL in *Dnmt3a*^Δ/Δ^ is consistent with the presence of mutations in DNMT3A in human T cell malignancies^21^. Our analysis of overexpressed and underexpressed genes in these hematologic malignancies shows a significant overlap with deregulated molecular events detected in human disease with 21% overexpressed and 11% underexpressed genes conserved between mouse and human CLL and even higher overlap with 39% overexpressed and 38% underexpressed conserved between mouse and human PTCL. Given that Dnmt3a is a methyltransferase whose direct consequence of inactivation would be expected to affect DNA methylation patterns we next focused on genes whose up-regulation was also accompanied by promoter hypomethylation. We identified 129 and 84 genes hypomethylated and overexpressed in CLL (HOC genes) and PTCL (HOT genes), respectively. 23 HOC genes were not only overexpressed in human CLL, with many of them also reported to be hypomethylated in human CLL^8^. Similarly, 26 HOT genes were overexpressed in human CD8+ PTCL but their methylation status have not been reported yet. Altogether, these data suggest that *EμSRα-tTA;Teto-Cre;Dnmt3a*^*fl/fl*^*;Rosa26LOXP*^*EGFP/EGFP*^model is a suitable model to study pathology of CLL and PTCL.

Since our data suggest that HOC and HOT genes might be targets of Dnmt3a it is also possible that their increased and untimely expression contribute to disease development. In CLL, *Zbtb32* (Zinc Finger and BTB Domain Containing 32 gene) was recently identified as a gene whose increased expression predicts whether patients without disease will develop CLL later in life^22^. In PTCL one of the candidate oncogenes is STAT1 (Signal transducer and activator of transcription 1), whose inappropriate activation has been observed in a variety of malignant cells from breast cancer, head and neck squamous carcinoma, melanoma, lymphoma and leukemia, suggesting that STAT1 may under specific conditions contribute to malignant transformation^23^. Such idea is further supported by findings that *Stat1*^*−/−*^ mice are partially protected from *v*-*abl*-induced leukemia development, suggesting that Stat1 functions as an oncogene^24^. However, only future functional studies will carefully dissect the potential contribution of these genes to the development of CLL and PTCL.

Lastly, recent studies using *Mx1-Cre* transgene to conditionally delete Dnmt3a in HSPCs followed by transplantation into lethally irradiated recipients showed that the vast majority of mice (69%) develop myeloid disorders such as MDS and AML with rare occurrences of CD4+ CD8+ double positive T-ALL or B-ALL^18^,^19^. Interestingly, we have not observed the development of myeloid malignancies in *EμSRα-tTA;Teto-Cre;Dnmt3a*^*fl/fl*^*;Rosa26LOXP*^*EGFP/EGFP*^ model, despite the fact that Dnmt3a – like in *Mx1 - Cre;Dnmt3a*^*fl/fl*^ model – is conditionally inactivated in HSPCs and all hematopoietic lineages. The differences in observed phenotypes could come from different experimental approaches in the sense that in *EμSRα - tTA;Teto - Cre;Dnmt3a*^*fl/fl*^*;Rosa26LOXP*^*EGFP/EGFP*^ deletion of Dnmt3a occurs in HSPCs of primary transgenic mice and bone marrow transplant of Dnmt3a-null HSPC into lethally irradiated recipients is not employed. Alternatively, we used FVB rather than C57BL/6 mice to inactivate Dnmt3a. As mouse strains differ in levels of gene expression, the Dnmt3a phenotypes may be strain specific. To address these possibilities directly we used *Dnmt3a*^Δ/Δ^ bone marrow isolated from *EμSRα-tTA;Teto-Cre;Dnmt3a*^*fl/fl*^*;Rosa26LOXP*^*EGFP/EGFP*^mice for adoptive transfer into lethally irradiated recipients. Interestingly, we observed the development of CLL with no signs of myeloproliferation, thus suggesting that Dnmt3a tumor suppressor function in prevention of CLL is autonomous to the hematopoietic system. This result also suggests that loss of Dnmt3a in the hematopoietic compartment may have strain-specific effects with FVB mice preferentially developing CLL and C57BL/6 mice preferentially developing myeloid malignancies. Further studies using *EμSRα-tTA;Teto-Cre; Dnmt3a*^*fl/fl*^*;Rosa26LOXP*^*EGFP/EGFP*^ in C57BL/6 mouse strain will further clarify this issue.

## Material and Methods

### Mouse Studies

*Dnmt3a*^Δ/Δ^ (*EμSRα-tTA;Teto-Cre;Dnmt3a*^*fl/fl*^*;Rosa26LOXP*^*EGFP/EGFP*^) and *Dnmt3a*^+/+^ (*EμSRα-tTA;Teto-Cre;Dnmt3a*^+/+^;*Rosa26LOXP*^*EGFP/EGFP*^) mice were generated as previously described^9^,^25^.

### FACS

FACS analysis was performed as described previously^11^. Briefly, B-1a cells were identified as being positive for B220, CD19, and CD5 cell surface markers, while CD8+ T cell were positive for CD8 and CD3 cell surface markers. Mice diagnosed with MBL had 2–20% B-1a cells in blood, mice diagnosed with CLL had >20% B-1a cells in blood, mice diagnosed with PTCL had >25% CD3+ CD8+ cells in the spleen. B cell subsets shown in Fig. 5c were FACS sorted from the spleen and bone marrow of 8-week old FVB/N mice using the following markers: splenic B-1a (B220+, CD19+, CD5+), splenic marginal zone B cells (CD23+, CD21-, IgM-low), splenic follicular B cells (CD23-, CD21-, IgM-high), bone marrow derived immature B cells (B220+, IgM+, IgD-) and bone barrow derived mature B cells (B220+, IgM+, IgD+). Analysis was performed at the UNMC Flow Cytometry Facility.

### Western Blot

Western blots were performed as previously described^12^ with use of the following antibodies: Dnmt3b (52A1018, Imgenex), Dnmt1 (H-300, Santa Cruz), and HDAC1 (ab7028, Abcam).

### Combined Bisulfite Restriction Analysis (COBRA)

COBRA analysis was carried out as described previously^12^,^17^. Primers used in this study are as follows:

Sgk3: TATTTTTTGTTTATAAATGTTGGTGG (F), AAACACTAAAAAAATAACATCATTCTC (R)

Nfam1: GGTTGAGTAAGAGTAAAGAAGATAGGTTAT (F), AACAAAAAAACAAACCCAAAAATAT (R)

Pstpip2: TGTATGTTTTTGGAGAGTAATAGGGT (F), TCCTTCCTAACAAAACCAATATCTT (R)

Plscr1: AGGAAATATAATAGAGGTGAAGATAGTAGG (F), CCATAACACTCCAATACTAAAAAAAA (R)

Crtam: AAGAGTTTTTATTGGGTTTTTATTTTT (F), ACTCACAAAAATCCACTTCCTAAATAC (R)

Sgk1: TGTTTTTATGGGTATTAAATGGTATA (F), TACAACCTAAATTTACTATCAAAATTACTT (R)

Samd3: TTGGAAGTTATTTTTGATATAGATTTTGT (F), TCACTTTCCAAAAAAACATAAACAC (R)

Oas3: TGGGGTTGTATAAAGGATTAAGGTA (F), ACACACACATACACAAAAATACAAACA (R)

Fam169b: TTGGAAATTGAATTTTAATTTATTAGGAT (F), AAAAAAACCACTCTTCAACCTATCA (R)

Pvt1: GGAGTTTTAAGTGGGATTTTTTAAA (F), CATACAATCACTCCCTAACAAAATAAA (R)

Dbi: GTAAAGGTAGGGTTAGGGTTGTTGT (F), TTCCTCTTTCAAAAATAACAAAAAAAA (R)

Il5ra: GGAGAATTTATGTTTTTTTAGAGTGTT (F), ATCATCCCATTAATCATTTATATTTTTATA (R)

Zbtb32: AAAGATTAGGTGTGTGTGATTTAGATTT (F), AAATTTCAAAAACTTTTTATCCCTTC (R).

### Whole genome Bisulfite sequencing (WGBS)

Splenic B-1a cells (EGFP+ CD5+ CD19+ B220+) were FACS sorted from Dnmt3a^+/+^ control (N = 1), and a *Dnmt3a*^Δ/Δ^ mouse with CLL (N = 1). Splenic CD8+ T cells (EGFP+ CD8+ CD3+) were FACS sorted from *Dnmt3a*^+/+^ control (N = 1), and a *Dnmt3a*^Δ/Δ^ mouse with PTCL (N = 1). Genomic DNA was isolated using standard protocols. The WGBS was performed in DNA Services facility at the University of Illinois at Urbana-Champaign, Roy J. Carver Biotechnology Center using two lanes for each sample on the Illumina HiSeq2500 sequencer with paired-end 160 bp reads. Each lane produced over 310 million reads. Sequence tags were aligned with the mouse genome (Dec. 2011 mus musculus assembly mm10, Build 38) using the methylated sequence aligner Bismark^26^ by the University of Nebraska Epigenomics Core facility. The resulting data file contains the percent methylation at each CpG measured. Each individual CpG was retained and percent methylation determined only if it was represented by ≥5 individual sequences. Correlation based, average linkage hierarchical clustering of genome location matching CpG methylation percentages per sample was performed using the R software package RnBeads^27^. Genome location matching differentially methylated cytosine (DMCs) and differentially methylated regions (DMRs) were determined using the R software package DSS^28^. DMCs were determined by first smoothing the raw percent methylation values based on a moving average algorithm and smoothing span of 500 bases. DMRs were then determined based on average DMC methylation change of 30% or greater, at least 50% or greater individual DMC P-values less than 0.05, minimum base pair length of 100, minimum of three DMCs represented, and the resulting DMRs were averaged if they were closer than 50 bases. DMRs were aligned with the mouse genomic repeats. Genomic repeats were acquired from the UCSC Genome table browser based on the RepeatMasker program^29^. The repeat was retained if the overlap between the DMR and repeat was more than 25 percent of the length of the repeat. WGBS data is available for download through the NCBI Gene Expression Omnibus (GSE78146).

### RNA-seq

RNA was isolated from FACS sorted splenic B-1a cells (EGFP+ CD5+ CD19+ B220+) from Dnmt3a^+/+^ control (N = 2), and *Dnmt3a*^Δ/Δ^ CLL (N = 8). Splenic CD8+ T cells (EGFP+ CD8+ CD3+) were FACS sorted from *Dnmt3a*^+/+^ control (N = 2) and *Dnmt3a*^Δ/Δ^ PTCL (N = 3). RNA was isolated as previously described^12^. Library generation was performed using the TruSeq mRNA kit. The resulting libraries were sequenced on the Illumina HiSeq 2000 platform using paired-end 100 bp runs (SeqMatic, Fremont, CA). The resulting sequencing data was first aligned using TopHat (version 1.0.0) and mapped to the Mus musculus UCSC mm10 reference genome using the TopHat 2 aligner. Cufflinks 2 was used to estimate FPKM of known transcripts, perform de novo assembly of novel transcripts, and calculate differential expression. For differentially expressed genes, we considered those genes with a fold change ≥2 and a q-value <0.05 to be significant. RNA-seq data is available for download through the NCBI Gene Expression Omnibus (GSE78146). RNA-seq data obtained from 5 normal human B cell samples and 15 human CLL tumors was downloaded from the Gene Expression Omnibus (GSE70830) and used to identify differentially expressed genes in human CLL (q < 0.05 was considered significant, as determined by Cufflinks).

### Microarray

Microarray data from 5 normal Tonsil T cells (GSE65135) were downloaded from NCBI Gene Expression Omnibus and compared to 15 cytotoxic PTCL samples^10^. Datasets were generated with Affymetrix U133 plus 2 arrays and analyzed using Affymetrix Expression Console and Transcriptome Analysis Console (v3.0). Data was analyzed using a one-way between-subject ANOVA to generate P-values and identify differentially expressed genes (P-value < 0.05 and fold change >1.5).

### Statistical Analysis

Comparison of overlap between mouse and human datasets was performed using a statistical simulation in which we randomly selected samples from the human genes list and mouse gene list without replacement. The number of times the sample human and mice genes overlapped was computed. This process ran for 100,000 times and for runs where the number of times the genes overlapped was greater or equal to the number observed it was counted. The P-value was computed by dividing the number of overlaps greater than that observed by 100,000. Continuous variables were compared using 2-sample Student’s *t* tests; results are presented as mean with error bars representing standard deviation. The Kaplan-Meier method was used to estimate disease-free survival distribution. All other statistical methods are described in the relevant section of the material and methods.

### Quantitative Real-Time qRT-PCR

qRT-PCR was performed as previously described^12^. Primer sequences used in experiments presented here are as follows:

Human Primers:

STAT1: ACAACCTGCTCCCCATGTCT (F), GCAGGAGGGAATCACAGATG (R)

JDP2: CCGGGAGAAGAACAAAGTCG (F), CGGTTCAGCATCAGGATGAG (R)

ZBTB32: GGAGACGCACTACCGAGTCC (F), GAAGGTGGAGCGGATGGTC (R)

ZBTB38: TGGAGGACTCAGAACCAAGG (F), CCGTGTCACTGTGAAAGTCG (R)

Mouse Primers:

Stat1: GGCCCCGAATTTGACAGTAT (F), ACCAGCAGTGCTCAGCAAAT (R)

Jdp2: GCCGGGAAAAGAACAAAGTC (F), GCGGTTGAGCATCAGGATAA (R)

Zbtb32:TCAGCCCTTGGCAGATAGAA(F), CACAGAGGGCATCGATAGGG(R)

Zbtb38: CGAGTGATTTCTGCCAAAGC (F), CACATAAGATGCCCCGAATG (R)

### Study approval

This study was performed in accordance with the guidelines established by the Guide for the Care and Use of Laboratory Animals at the National Institutes of Health. All experiments involving mice were approved by the IACUC (Protocol number: 08-083-10-FC) at the University of Nebraska Medical Center. The Institutional Review Board of UNMC approved the human studies, and patients provided informed consent prior to the study in accordance with the Declaration of Helsinki.

## Additional Information

**How to cite this article**: Haney, S. L. *et al*. Loss of Dnmt3a induces CLL and PTCL with distinct methylomes and transcriptomes in mice. *Sci. Rep.* **6**, 34222; doi: 10.1038/srep34222 (2016).

## Supplementary Material

Supplementary Information

Supplementary Table S1

Supplementary Table S2

Supplementary Table S3

Supplementary Table S4

Supplementary Table S5

Supplementary Table S6

Supplementary Table S7
