# Supplementary material for: Loss of Dnmt3a induces CLL and PTCL with distinct methylomes and transcriptomes in mice
Source: Sci Rep. 2016 Sep 28;6:34222. doi: 10.1038/srep34222 (PMC5039761; doi:10.1038/srep34222)
Supplement: Supplementary Information [file srep34222-s1.pdf]

Supplementary Information for:

Loss of Dnmt3a induces CLL and PTCL with distinct methylomes and transcriptomes in mice.

Staci L. Haney[1*], Garland M. Upchurch[2*], Jana Opavska[2], David Klinkebiel[3], Adams Kusi Appiah[4], Lynette M. Smith[4], Tayla B. Heavican[5], Javeed Iqbal[5,6], Shantaram Joshi[1,6], Rene Opavsky[1,2,6,#]

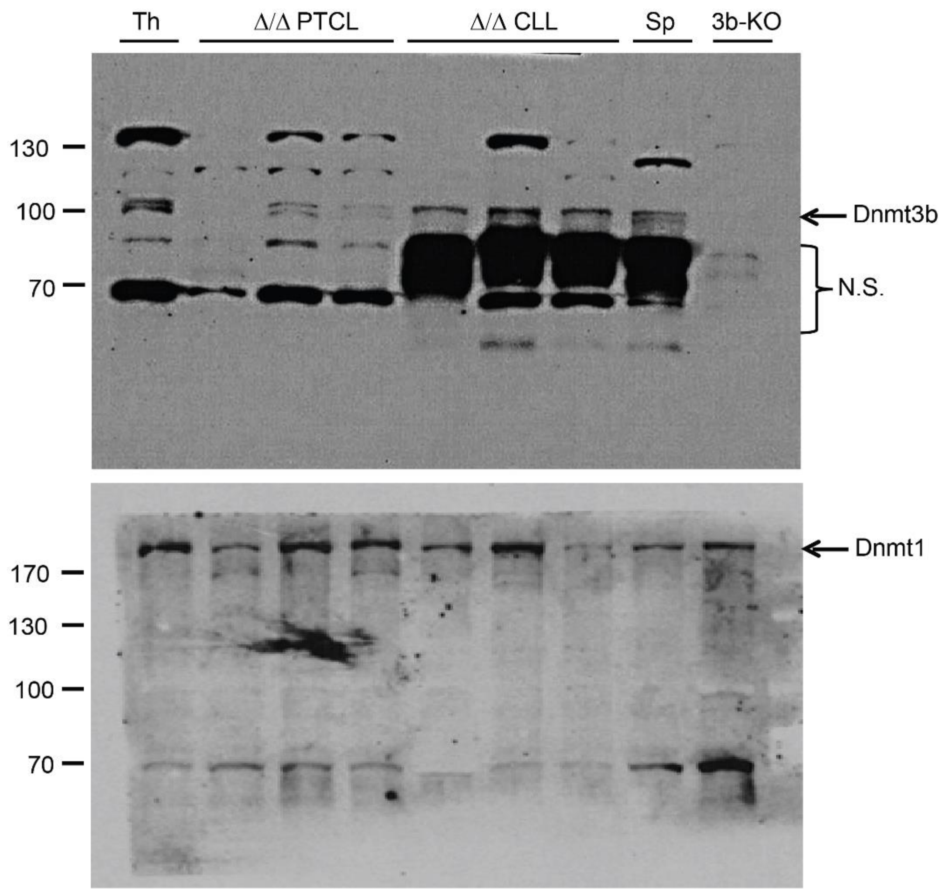

Supplementary Figure 1. Full length immunoblots as presented in Figure 1b. Immunoblot analysis of Dnmt3b and Dnmt1 proteins in *Dnmt3a*$^{+/+}$ normal thymus (Th), *Dnmt3a*$^{+/+}$ normal spleen (Sp), *Dnmt3a*$^{\Delta/\Delta}$ PTCL, and *Dnmt3a*$^{\Delta/\Delta}$ CLL samples. *Dnmt3b*$^{-/-}$ (3b KO) T cell lymphoma line was used as a negative control for Dnmt3b immunoblot. The location of Dnmt3b and Dnmt1 proteins are marked by arrows. Non-specific bands (N.S.) present in the Dnmt3b KO control are labelled.

a

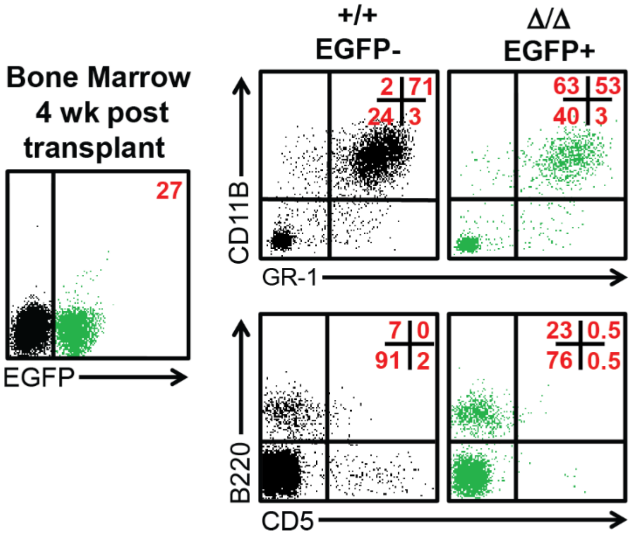

b

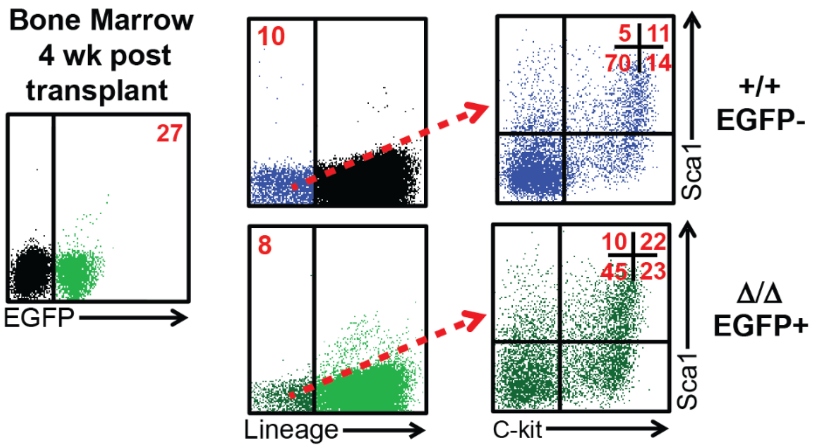

Supplementary Figure 2. Dnmt3a's tumor suppressor function is cell autonomous to the hematopoietic system. (a) FACS diagram showing CD11b and GR-1 expression (top) and B220 and CD5 expression (bottom) in EGFP- (black) and EGFP+ (green) cells isolated from the bone marrow of a lethally irradiated FVB recipient mice injected with *Dnmt3a*$^{\Delta/\Delta}$ bone marrow. The mouse was harvested 4 weeks post injection. Percentage of cells staining positive in each quadrant are shown in red. **(b)** FACS diagram showing percentage of lineage-Sca1+ckit- cells in EGFP- (blue) and EGFP+ (green) cells isolated from the bone marrow of a lethally irradiated FVB recipient mice injected with *Dnmt3a*$^{\Delta/\Delta}$ bone marrow. The mouse was harvested 4 weeks post injection. Percentage of cells staining positive in each quadrant are shown in red.

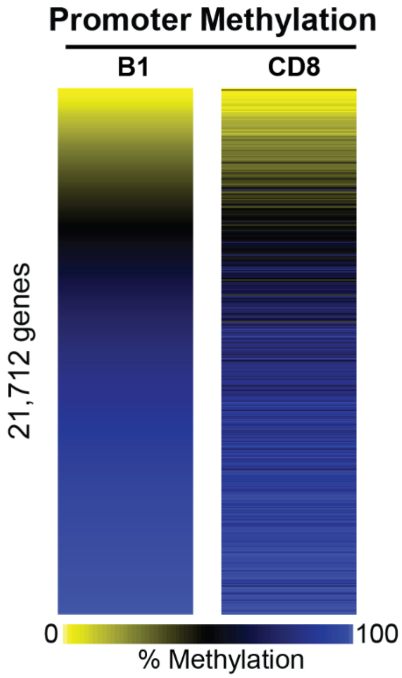

Supplementary Figure 3. Methylome of long promoter regions in B-1a and CD8. Methylation status of 21,838 promoters in B-1a and CD8 samples as determined by WGBS. Methylation percentage for individual CpGs were annotated to the promoter regions -1,500bp to +500bp relative to the transcription start site. Methylation percentages for all CpGs across the 2000bp region were averaged to give a mean methylation value for each gene promoter. Hypomethylation is shown in yellow and hypermethylation in blue.

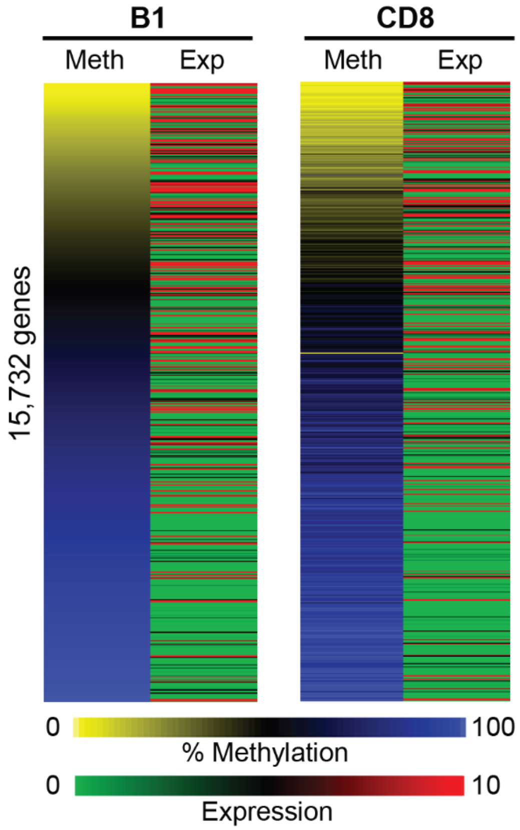

Supplementary Figure 4. Methylome of long promoter regions with corresponding gene transcription in B-1a and CD8. Heat map presentation of 2,000bp promoter methylation (analyzed as in Figure S3) and corresponding gene expression (presented as average FPKM values as determined by RNA-seq) in mouse splenic B1 and CD8 cells for 15,732 genes. Genes with high FPKM values are shown in red and genes with low FPKM values are shown in green. Heat maps are organized in the same gene order to match data for methylation and gene expression.

B-1 Highest Expressed Genes	
**Physiological System Development and Function**	**# Genes**
Hematological system development and function	994
Tissue Morphology	927
Organismal survival	1251
Hematopoiesis	619
Lymphoid tissue structure and development	571
**Pathways**	**# Genes**
EIF2 signaling	132
Protein Ubiquitination pathway	155
Glucocorticoid receptor signaling	155
Regulation of eIF4 anf p70S6K signaling	100
CD28 signaling in T helper cells	84
**Diseases and Disorders**	**# Genes**
Infectious disease	956
Organismal injury and abnormalities	1108
Inflammatory response	616
Immunological disease	1037
Cancer	1011

CD8 Highest Expressed Genes	
**Physiological System Development and Function**	**# Genes**
Organismal survival	1151
Hematological system development and function	903
Tissue Morphology	822
Hematopoiesis	563
Lymphoid tissue structure and development	498
**Pathways**	**# Genes**
EIF2 signaling	130
Protein Ubiquitination pathway	157
Regulation of eIF4 anf p70S6K signaling	96
Mitochondrial dysfunction	101
Glucocorticoid receptor signaling	141
**Diseases and Disorders**	**# Genes**
Infectious disease	842
Organismal injury and abnormalities	1525
Inflammatory response	526
Cancer	1427
Hematological disease	840

Supplementary Figure 5. Summary of Ingenuity Pathway analysis (IPA) of all highly expressed genes (FPKM ≥ 10) in B-1a and CD8 control samples. P-values were less than 0.05 for all categories.

B-1 specific Genes	
**Physiological System Development and Function**	**# Genes**
Hematological system development and function	127
Tissue Morphology	107
Humoral immune response	75
Hematopoiesis	82
Lymphoid tissue structure and development	81
**Pathways**	**# Genes**
B Cell Receptor Signaling	22
PI3K Signaling in B Lymphocytes	17
Role of NFAT in Regulation of the Immune Response	17
Primary Immunodeficiency Signaling	9
FcRIIB Signaling in B Lymphocytes	8
**Diseases or Functions Annotation**	**# Genes**
Quantity of leukocytes	83
Proliferation of B lymphocytes	40
Quantity of mononuclear leukocytes	71
Quantity of lymphocytes	69
Quantity of immunoglobulin	42

CD8 Specific Genes	
**Physiological System Development and Function**	**# Genes**
Hematological system development and function	62
Immune cell trafficking	36
Digestive system development and function	8
Hepatic system development and function	3
Organ development	20
**Pathways**	**# Genes**
Role of IL-17A in Psoriasis	2
S-methyl-5'-thioadenosine Degradation II	1
Leukotriene Biosynthesis	2
Mismatch Repair in Eukaryotes	2
D-myo-inositol (1,4,5)-trisphosphate Degradation	2
**Diseases or Functions Annotation**	**# Genes**
Activation of leukocytes	24
Function of oval cells	3
Insulin-dependent diabetes mellitus	21
Systemic autoimmune syndrome	37
Cytolysis of lymphocytes	8

Supplementary Figure 6. Summary of Ingenuity Pathway analysis (IPA) of those genes specifically expressed in either B-1a or CD8, but not the other. Genes with an FPKM ≥ 10 were used for analysis. P-values were less than 0.05 for all categories.

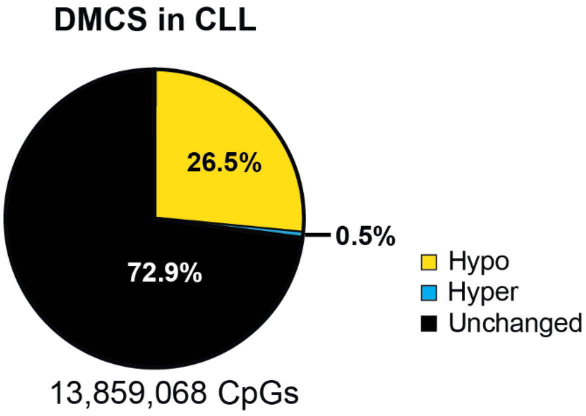

Supplementary Figure 7. Differentially methylation cytosines (DMCS) in CLL and PTCL. Methylation status of 13,859,068 CpGs in *Dnmt3a*$^{\Delta/\Delta}$ CLL relative B-1a (left) and *Dnmt3a*$^{\Delta/\Delta}$ PTCL relative to CD8+ T cells (right). Hypomethylated (yellow) and hypermethylated (blue) CpGs had a 30% or greater decrease or increase in methylation, respectively. CpGs whose methylation was unchanged are shown in black.

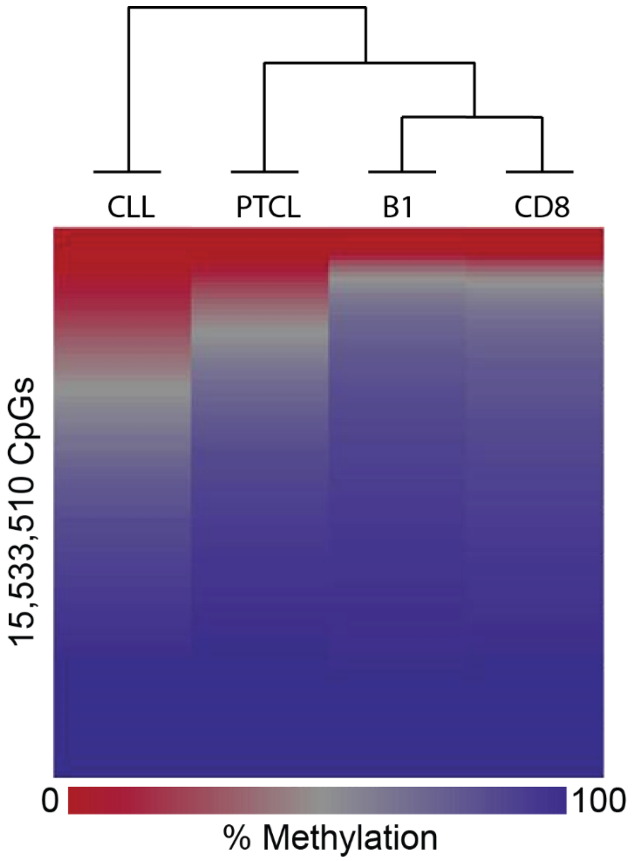

Supplementary Figure 8. Methylation status of 15,533,510 CpG dinucleotides in control B-1a, control CD8, and Dnmt3a-deficient CLL and PTCL cells as determined by whole-genome bisulfite sequencing (WGBS). Hypomethylated CpGs are shown in red whereas hypermethylated CpGs are in blue. Hierarchical clustering of samples derived from WGBS datasets are shown at the top.

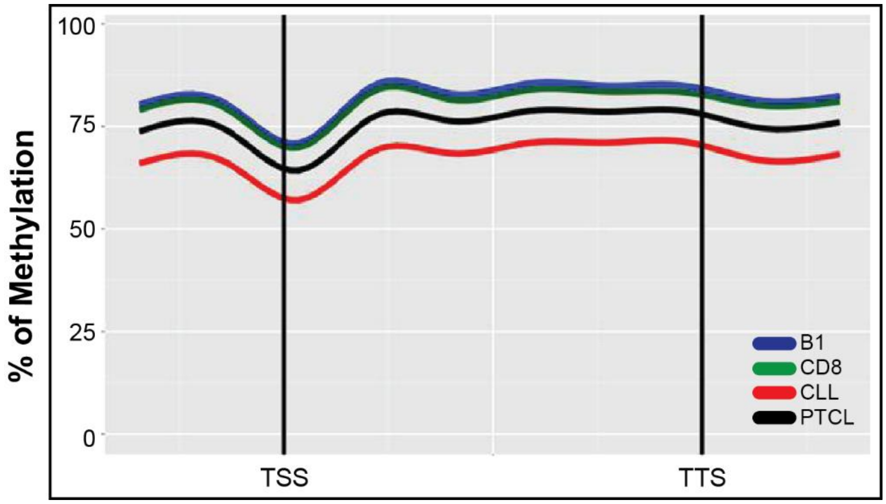

Supplementary Figure 9. Average CpG methylation percentage for B-1a (blue), CD8 (green), *Dnmt3a*$^{\Delta/\Delta}$ CLL (red), and *Dnmt3a*$^{\Delta/\Delta}$ PTCL (black) relative their position within genes. Location of transcription start site (TSS) and the transcription termination site (TTS) are shown.

PTCL	
**Physiological System Development and Function**	**# Genes**
Hematological System Development & Function	429
Tissue Morphology	399
Organismal Survival	489
Hematopoiesis	246
Lymphoid Tissue Structure & Development	243
**Diseases and Disorders**	**# Genes**
Immunological Disease	470
Endocrine System Disorders	307
Gastrointestinal Disease	117
Metabolic Disease	234
Cancer	1444
**Inhibited Pathways**	**# Genes**
TNFR1 Signaling	15
TNFR1 Signaling	11
iNOS signaling	13
RANK signaling in osteoclasts	18
CD18 Signaling in T Helper cells	21
**Activated Pathways**	**# Genes**
ATM signaling	22
Estrogen-mediated S-phase Entry	11
Cyclins and Cell Cycle Regulation	17
Apoptosis signaling	17
PDGF Signaling	14

CLL	
**Physiological System Development and Function**	**# Genes**
Hematological System Development & Function	346
Tissue Morphology	297
Immune Cell Trafficking	222
Hematopoiesis	179
Lymphoid Tissue Structure & Development	172
**Diseases and Disorders**	**# Genes**
Immunological Disease	340
Endocrine System Disorders	230
Gastrointestinal Disease	769
Metabolic Disease	177
Inflammatory Response	332
**Inhibited Pathways**	**# Genes**
iSOC-iCOSL Signaling in T Helper Cells	22
Calcium-induced T Lymphocyte Apoptosis	13
p53 signaling	14
G2/M DNA Damage checkpoint Regulation	9
Mouse Embryonic Stem Cell Pluripotency	12
**Activated Pathways**	**# Genes**
Pattern Recognition Receptors for Bacteria/Viruses	22
Toll-like Receptor Signaling	15
IL-6 Signaling	17
Estrogen-mediated S-phase Entry	6
Signaling by Rho Family GTPases	24

Supplementary Figure 10. Summary of Ingenuity Pathway analysis (IPA) of all differentially expressed genes (FPKM ≥ 10) in *Dnmt3a*$^{\Delta/\Delta}$ PTCL and *Dnmt3a*$^{\Delta/\Delta}$ CLL relative to control samples. Genes with a fold change ≥ 2 and a q-value < 0.05 were used in the analysis. P-values were less than 0.05 for all categories.

Fold change	Mut % Meth	B1 % Meth	Gene
4.5	36	87	Prc1
8.2	26	78	Prkar2b
2.7	6	85	Pstpip2
2.1	19	71	Ptms
2.0	20.5	76.5	Ptp4a3
2.9	13	80	Pvt1
3.5	16	71	Racgap1
2.6	11	83.5	Rbm47
3.8	20	84	Rdh12
2.9	20	81	Rtn4ip1
5.3	3	67	S100a4
2.2	15	69	Sgk3
6.6	31	82	Sirt2
2.0	14	82	Slc37a2
3.1	20	90	Slc39a4
2.3	19	79	Slc7a7
2.2	13	76	Snx20
2.3	33	86	Soat1
2.5	10	71	Sp110
7.0	15	65	Spire1
4.7	9	65	Sspn
2.0	13	66	St3gal2
2.7	13	89	St3gal6
2.5	12	75	Stx7
2.8	7	59	Tagln2
2.1	17.5	91	Ticam1
4.6	2	63	Ticam2
2.9	9	62	Tlr2
7.7	5	69	Tmem106a
2.0	9	90	Tmem229b
5.3	32	90	Tnf
2.2	22	87	Tnfrsf13b
2.6	23	88	Tns1
2.3	8	82	Txn2
2.4	5	73	Ubash3b
5.2	2	59	Uhrf1
12.6	17	84	Upb1
2.3	4	50	Ypel2
22.2	9	58	Zbtb32
2.2	19.5	81.25	Zbtb38
5.6	35	91	Zcchc14
4.7	16	66	Zcchc18
3.3	15	70.5	Zeb2

Fold change	Mut % Meth	B1 % Meth	Gene
13.1	12	70	Gas7
379.0	14	82.5	Gnb3
32.1	21	78	Gpm6a
3.9	9	87	H6pd
4.8	13	77	Hepacam2
2.8	10	64	Hmga2-ps1
3.5	16	90	Hpse
15.1	19	75	Ifitm6
2.3	27	91	Ift27
2.6	9	69	Igsf8
4.0	11	81	Il5ra
3.0	20	86	Il9r
2.2	25	84	Inppl1
2.9	9	97	Irgm2
33.9	15	71	Itgam
2.7	15	83	Itgb7
2.7	31	91	Itpr1
3.1	27	82	Itsn1
2.1	11	84	Krt222
6.0	16	86	Lmna
2.8	11	91	Lsp1
2.8	9	85	Ly6c2
2.8	29	89	Man1c1
4.5	7	94	Mgmt
2.2	15.5	83.5	Mtss1
17.9	19	81	Myadm
2.0	8	71	N4bp3
2.5	18	85	Ncf4
4.1	26	96	Neurl1a
6.5	11	84.5	Nfam1
2.1	14	65	Nfkbiz
3.1	8	75	Nrp2
10.0	23	84	Ntng2
10.5	10	59	Ociad2
2433.2	9	67	Olfml2a
4.7	19	87.5	Pdcd1lg2
2.5	35	90	Pfkfb3
30.0	6	87	Pik3r6
2.9	27	89	Plec
4.4	11	76	Plscr1
4.0	17	80	Pon3
8.3	26	89	Ppfia4
2.8	9	60	Ppil1

Fold change	Mut % Meth	B1 % Meth	Gene
2.9	15.5	78.5	1810046K07Rik
8.6	19	87	2700054A10Rik
4.0	19	86	2810417H13Rik
2.4	12	65	Abi3
7.4	26	90	Adamtsl4
17.7	28	88	AF067061
6.7	22	91	Ahnak
105.9	10	72	AI427809
5.3	13	87	Aldh3b1
2.1	7	60	Anxa11
12.8	3	58	Anxa3
2.9	3	66	Apobec3
4.8	1	65	Arap3
3.5	20	81	Arhgap33
2.2	28	79	Arid3b
3.5	20	93	Aurkb
93.2	20	83	Avil
2.8	38	88	BC064078
2.4	27	80.5	Blvrb
8.5	21	74	C2
2.7	4	77	Capn2
18.3	10	88	Cd300ld
2.8	16	75.333	Cd80
4.8	18	85	Cdc42ep4
2.4	8	65	Cdca7
2.9	2	56	Cisd3
2.2	20	93	Cnp
5.3	13	78	Crip1
8.1	25	90	Cyp11a1
2.7	1	49	Dbi
8.6	5	61	Dgkg
3.4	10	82	Dse
3.7	10	58	E2f2
2.6	19	79	Ebi3
2.0	19	82	Ece1
2.4	32	88	Eps8
7.3	20	86	Espn
5.0	16	82	Etv5
2.7	24	86	Evi5
19782.5	18	76	Fabp7
2.2	35	95	Fam46c
2.5	12	77	Fdps
3.3	15.5	81	Fgd2

Supplementary Figure 11. Genes Hypomethylated and Overexpressed in *Dnmt3a*$^{\Delta/\Delta}$ CLL (HOC genes). List of HOC genes. Percentage of promoter methylation in B-1a (B1 % meth; blue), and *Dnmt3a*$^{\Delta/\Delta}$ CLL (CLL % meth; yellow) is shown within boxes. Similarly, fold differences in gene expression between *Dnmt3a*$^{\Delta/\Delta}$ CLL relative to B-1a (B1 vs Mut; red) is shown within boxes.

Exp	PTCL % Meth	CD8 % Meth	Gene	Exp	PTCL % Meth	CD8 % Meth	Gene	Exp	PTCL % Meth	CD8 % Meth	Gene
5.4	12	87	Per3	4.9	11.5	77.5	Ifi47	7.6	20	79	1700025G04Rik
31.7	27	74	Pif1	3.5	8	83	Ifitm10	14.2	6	51	1700048O20Rik
22.1	6	73	Plac8	2.3	10	94	Ikzf3	10.4	20	77	4921525O09Rik
2.8	10	71	Plekha8	3.1	26	85	Il18rap	3.8	12	76	Abi3
6.0	4	55	Ppil1*	2.8	10	88	Il2rb	3.4	9	69	Acot7
3.1	23	95	Pvt1*	3.3	10	74	Impa1	5.2	11	71	Alpk3
2.0	13	68	Racgap1	9867.8	21	84	Islr	6.8	12	87	Amica1
2.0	22	82	Ran	5.4	21	85	Jdp2	11.3	7	70	Apobr
3.4	14	63	Reep5	3.2	31	86	Keap1	10.2	8.5	81.5	Arl4d
3.9	8	92	Rnasel	5.9	7	77	Klrc1	4.5	1	81	Atp8b4
5.4	8	82	Rnf43	2.7	10	80	Klrd1	4.7	11	78	AW112010
9.2	10	82	Samd3	22.9	7.5	85	Klre1	2.7	6	75	B4galt5
3.2	37	85	Sh2d1a	10.7	18	85	Lpar5	2.6	9	86	C920025E04Rik
2.0	22	90	Sh2d3c	4.6	5	53	Lyrm9	28.9	2	72	Ccr2
3.3	19	85	Sla	22.8	22	86	Mmp14	2.4	28	83	Celsr1
2.2	37	90	Sntb2	13.7	12	86	Ms4a4b	3.6	7	70	Cln3
8.6	14	85	Stat1	8.0	50	98	Ms4a4c	4.6	12.5	82	Coro2a
7.7	2	68	Tmem37	2.5	23	91	Myo1c	3.2	10	74	Crtam
2.3	5	64	Tmpo	8.2	7	61	Myo6	6.6	9	81	Cxcr5
3.2	34	88	Trim14	4.6	34	91	N4bp1	4.6	6	62	Fah
17.6	7	74	Ttll11	3.1	4	61	Nfe2l2	3.5	50	94	Fcgrt
2.1	21	87	Txk	2.4	31	96	Nkg7	12.2	7	88	G0s2
2.0	8	75	Uba7	2.6	13	76	Nod1	5.4	12	67	Gdf11
8.2	3	70	Wfikkn2	23.6	42	91	Oas2	3.3	2	64	Gimap4
2.0	30	81	Wipf1	12.0	14	82	Oas3	3.1	3	70	Gimap7
2356.8	17	75.5	Wnt8a	17.8	17	78	Osbpl3	9.3	19	66	Gm19705
4.7	25	74	Xlr3b	2.1	30	84	Oxr1	1437.6	4	71	Gpnmb
3.3	11	70	Zfp808	5.0	19	86	P2rx7	3.4	6	79	Gzmm

Supplementary Figure 12. Genes Hypomethylated and Overexpressed in *Dnmt3a*$^{\Delta/\Delta}$ PTCL (HOT genes). List of HOT genes. Percentage of promoter methylation in CD8 (CD8 % meth; blue), and *Dnmt3a*$^{\Delta/\Delta}$ PTCL (PTCL % meth; yellow) is shown within boxes. Similarly, fold differences in gene expression between *Dnmt3a*$^{\Delta/\Delta}$ PTCL relative to CD8 (CD8 vs Mut; red) is shown within boxes. Genes common between HOC and HOT datasets are shown in red*.
